# The Gut Connection: Exploring the Possibility of Implementing Gut Microbial Metabolites in Lymphoma Treatment

**DOI:** 10.3390/cancers16081464

**Published:** 2024-04-11

**Authors:** Ahmad K. Al-Khazaleh, Dennis Chang, Gerald W. Münch, Deep Jyoti Bhuyan

**Affiliations:** 1 NICM Health Research Institute, Western Sydney University, Penrith, NSW 2751, Australia; d.chang@westernsydney.edu.au; 2 Pharmacology Unit, School of Medicine, Western Sydney University, Campbelltown, NSW 2560, Australia; g.muench@westernsydney.edu.au; 3 School of Science, Western Sydney University, Penrith, NSW 2751, Australia

**Keywords:** lymphoma, Hodgkin’s lymphoma, non-Hodgkin’s lymphoma, Burkitt lymphoma, gut microbiota, short-chain fatty acids, gut metabolites, postbiotics

## Abstract

**Simple Summary:**

Gut microbiota dysbiosis may contribute to lymphoma by disrupting gut microbial metabolites. Some gut microbial metabolites can cause chronic inflammation, increasing the risk of lymphoma, while others have shown promise to prevent it in preclinical studies. For instance, short-chain fatty acids and urolithin A have shown immunomodulatory and antiproliferative properties against lymphoma cell lines in vitro. Further research is needed to understand the significance of gut microbial metabolites in lymphoma and explore their potential therapeutic applications.

**Abstract:**

Recent research has implicated the gut microbiota in the development of lymphoma. Dysbiosis of the gut microbial community can disrupt the production of gut microbial metabolites, thereby impacting host physiology and potentially contributing to lymphoma. Dysbiosis-driven release of gut microbial metabolites such as lipopolysaccharides can promote chronic inflammation, potentially elevating the risk of lymphoma. In contrast, gut microbial metabolites, such as short-chain fatty acids, have shown promise in preclinical studies by promoting regulatory T-cell function, suppressing inflammation, and potentially preventing lymphoma. Another metabolite, urolithin A, exhibited immunomodulatory and antiproliferative properties against lymphoma cell lines in vitro. While research on the role of gut microbial metabolites in lymphoma is limited, this article emphasizes the need to comprehend their significance, including therapeutic applications, molecular mechanisms of action, and interactions with standard chemotherapies. The article also suggests promising directions for future research in this emerging field of connection between lymphoma and gut microbiome.

## 1. Introduction

Cancer continues to pose a significant global health burden, with lymphoma being a type of cancer that affects the lymphatic system. Lymphoma comprises Hodgkin (HL) and non-Hodgkin (NHL) subtypes, with the latter being more prevalent and exhibiting higher incidence rates in developed countries. In 2020 alone, the NHL accounted for over 500,000 new cases and 250,000 deaths worldwide, underscoring its substantial morbidity and mortality [1]. Standard treatment modalities for lymphoma include chemotherapy, radiation therapy, and immunotherapy, which can facilitate remission and extend the survival of affected individuals [2]. However, current therapies against lymphoma face several shortcomings that limit their effectiveness in achieving long-term remission [3]. One significant limitation is the development of resistance to chemotherapy and targeted therapies. Lymphoma cells can acquire genetic mutations or activate alternative signaling pathways, leading to treatment resistance and disease relapse [4]. Another challenge is the toxicity associated with conventional therapies, which can cause severe side effects and impact the quality of life of patients [4]. Additionally, these treatments lack specificity in targeting cancer cells that often damage healthy tissues and organs. Immunotherapies, such as checkpoint inhibitors and CAR-T cell therapies, have shown promise in treating lymphoma; however, these therapies are not effective in all patients, and some individuals may experience adverse events [5]. These limitations highlight the need for continued research and development of novel therapeutic strategies to improve the treatment outcomes of lymphoma.

The gut microbiota, a diverse community of microorganisms residing in the gastrointestinal tract, has emerged as a critical player in human health and disease, including lymphoma [6]. Disruption in the composition of the gut microbiota, known as dysbiosis, can arise from various factors such as antibiotic use, diet, and lifestyle and has been linked to the development of lymphoma [6,7]. Dysbiosis can alter the production of gut microbial metabolites, compounds that influence host physiology.

Among the gut microbial metabolites, short-chain fatty acids (SCFAs) are generated through the fermentation of dietary fiber by the gut microbiota and have been observed to promote regulatory T-cell function, suppressing inflammation and potentially preventing lymphoma development [8]. In contrast, other gut microbial metabolites, such as lipopolysaccharides (LPS), can promote inflammation and increase the risk of lymphoma. Dysbiosis can lead to the release of LPS into the bloodstream, activating the immune system and fostering chronic inflammation, thereby heightening the lymphoma risk [8].

Emerging evidence suggests that gut microbial metabolites, including SCFAs, inosine, urolithin A, urolithin B, and bacteriocins, possess immunomodulatory and anticancer properties. Previous studies have demonstrated their potential therapeutic applications in various cancer types, including lymphoma [9]. However, the precise mechanisms by which the gut microbiota and its metabolites influence lymphoma risk and progression are still being elucidated, necessitating further research to identify therapeutic targets. This review aims to explore the relationship between gut microbiota and lymphoma, focusing on the role of gut microbial metabolites [9,10,11].

## 2. Lymphoma

**Figure 1:**
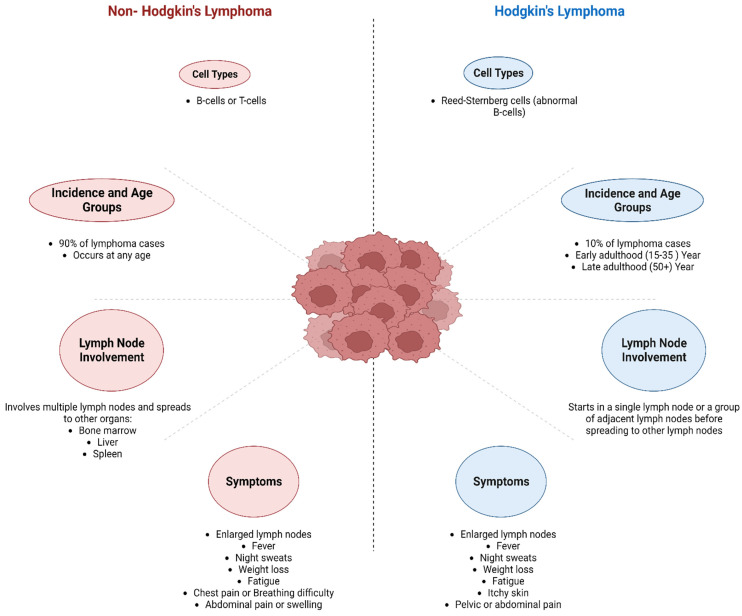
Key differences between Hodgkin’s lymphoma and non-Hodgkin’s lymphoma.

Lymphoma is a type of cancer that affects the lymphatic system, which is a part of the immune system. It can be categorized into two types: Hodgkin (HL) and non-Hodgkin (NHL), with prevalence rates of 10% and 90%, respectively. HL is further classified into classical and nonclassical lymphoma (Figure 1), whereas NHL is classified into B-cell, T-cell, and natural killer (NK) cell types. The symptoms of lymphoma can vary depending on the type and stage of cancer but may include swollen lymph nodes, fatigue, fever, weight loss, night sweats, and itching [2]. Treatment for lymphoma includes chemotherapy, radiation therapy, immunotherapy, or a combination of these approaches. The outlook for people with lymphoma varies depending on the type and stage of cancer. However, many people can achieve remission and lead long, healthy lives with appropriate treatment [12]. For clinical considerations, it is important to note whether a particular lymphoma is aggressive (high-grade) or indolent (low-grade). Except for nodular lymphocyte-predominant HL, the majority of indolent lymphomas belong to the category of NHL [2]. Indolent lymphomas have traditionally been considered less harmful if left untreated, but they are also more challenging to treat [2]. While this may appear paradoxical, it is due to the decreased growth rate of indolent tumors, which renders them less vulnerable to treatment [2]. The aggressiveness or indolence of the lymphoma and the performance status of patients determine whether therapy is curative, with survival as the aim, or palliative, with quality of life as the goal [2].

## 3. Classification of Lymphoma

### 3.1. Hodgkin’s Lymphoma (HL)

HL was initially reported in 1832, but the nature of the pathognomonic Reed–Sternberg cell used to diagnose the illness has only recently been revealed. Since the 1940s, radiotherapy has been employed to treat localized illness, and in the 1960s, successful combination chemotherapy regimens for anatomically advanced diseases were launched [13]. Throughout the last three decades, the result of HL has improved to the point that it is now one of the most treatable non-cutaneous malignancies [13]. With increased survival and longer follow-up, the significance of treatment-induced late effects has become clear, and contemporary therapeutic techniques must adequately account for these consequences [13].

HL, short for Hodgkin’s lymphoma, is a prevalent form of lymphoma in Western countries, with an annual occurrence rate of approximately three cases per 100,000 individuals [14]. It primarily impacts peripheral lymph nodes and organs like the liver, lung, and bone marrow. Based on histological features and the phenotype of tumor cells, HL is categorized into subtypes, including nodular sclerosis, mixed cellularity, lymphocyte-rich, lymphocyte-depleted, and nodular lymphocyte-predominant HL (NLPHL) [14]. Classical Hodgkin’s lymphoma (HL) comprises the aforementioned categories. HL cells are relatively rare, constituting only about 0.1–2% of the cells in the affected tissue [14]. In classical HL, the malignant cells are referred to as Hodgkin and Reed–Sternberg cells, whereas in the case of NLPHL, they are called lymphocyte-predominant (LP) cells [14]. These malignant cells are characterized by their large size, and in classical HL, they can be further classified as mononucleated Hodgkin cells or bi- or multinucleated Reed–Sternberg cells [14]. HL is a relatively rare cancer characterized by Reed–Sternberg cells and abnormal white blood cells in lymph nodes [15]. Moreover, Reed–Sternberg cells, which display a distinctive “owl’s eye” appearance and contain prominent nucleoli, represent a pathognomonic feature of HL [15]. These large, multinucleated cells can be readily identified in lymph node biopsies [15]. The cells are named in honor of Dorothy Reed and Carl Sternberg, the pioneering pathologists who initially described them. Although the absence of Reed–Sternberg cells in a biopsy does not preclude a diagnosis of HL, their presence remains a critical diagnostic criterion for this malignancy [15]. Epstein–Barr virus (EBV) infects tumor cells in around 40% of instances of classical HL [14].

### 3.2. Non-Hodgkin Lymphoma (NHL)

NHL is a malignant condition that arises from immune cells and manifests mostly as lymphadenopathy or solid tumors [16]. NHL is a more common type of lymphoma that can arise from any lymphocyte, a type of white blood cell, and can occur in any part of the body where lymphatic tissue is found [2]. NHL categorization is complicated and ever-changing, with more than 50 distinct subtypes mentioned in the most recent World Health Organization classification [16]. Non-specialists, on the other hand, can benefit by categorizing them as low-grade (indolent) or high-grade (aggressive) lymphoma, since this broad distinction indicates the likely natural course and care of the disease [16].

NHL is the sixth most prevalent cancer in Australia and the first among young people [12]. The International Agency for Research on Cancer reported that over 500,000 NHL cases were diagnosed globally in 2020 [1]. The highest incidence rates of lymphoma are found in Australia/New Zealand, Northern America, and Europe, and it is more prevalent in men than women [1]. However, the considerable differences in data reporting quality internationally make these statistics challenging to assess. Individual subtypes vary by geography, with follicular lymphoma being more prevalent in Western nations, T-cell lymphoma being more common in Asia, and EBV-linked (endemic) Burkitt lymphoma being more common in Africa [17]. Follicular lymphoma is the most frequent indolent lymphoma, while diffuse large B cell lymphoma (DLBCL) is the most common aggressive type [16].

Most NHL are caused by mature B lymphocytes, with a small percentage linked to T lymphocytes or natural killer (NK) cells. Furthermore, lymphoma occurs due to the gradual accumulation of DNA abnormalities such as gene mutation, amplification or deletion, and chromosomal translocation [18]. Certain lymphoma subtypes are associated with distinct acquired genetic abnormalities, such as translocation of the BCL2 oncogene in follicular lymphoma or translocation of the MYC oncogene in Burkitt lymphoma [18].

Infections such as EBV, *Helicobacter pylori*, and hepatitis C virus have also been linked to some subtypes of NHL [19]. NHL is more frequent in immunocompromised people, such as HIV/AIDS patients or organ transplant recipients [20]. Although smoking has been linked to specific lymphoma subtypes [20], is not a well-established risk factor for NHL [21]. Although a slightly increased risk among family members has been observed, NHL is considered nonhereditary.

#### Burkitt Lymphoma (BL)

Burkitt lymphoma (BL) is a highly aggressive type of NHL most commonly found in children in Africa, but also occurs in other parts of the world [22]. BL is characterized by the translocation of the MYC gene on chromosome 8 to one of the immunoglobulin loci on chromosomes 2, 14, or 22, resulting in the overexpression of MYC. This leads to uncontrolled cell growth and proliferation of B cells [22]. The standard treatment for BL combines chemotherapy and immunotherapy, which has a high success rate of 90% for patients who receive appropriate treatment [22]. However, the prognosis for BL depends on various factors, such as age, disease stage, and other medical conditions. In recent years, several advancements in understanding the molecular mechanisms underlying Burkitt lymphoma (BL) have been made, which have led to the development of targeted therapies for the disease [22]. One important aspect of BL is its association with the EBV, which produces a protein called Epstein–Barr nuclear antigen 1 (EBNA1) in all EBV-associated malignancies. EBNA1 is a dimeric viral protein that plays multiple roles in the pathogenesis of BL. BL is a frequent latency I cancer, and the infected cells might enter immunologically inactive latency programs. EBNA1 is exclusively expressed in this way to allow the EBV episome to be disassociated and maintained in dividing B cells [23,24].

Peyton Rous, a Nobel Laureate, investigated the early relationship between cancer and viral infections in chickens in 1910 [25]. Many viruses with carcinogenic mechanisms have been found since then. So far, eight oncogenic viruses (both RNA and DNA viruses) have been discovered to cause cancer via distinct pathways [26]. Oncogenic viral infections are responsible for 15% to 20% of all human cancers [27]. EBV is a herpesvirus (officially known as Human Gamma herpesvirus 4) commonly transmitted through bodily fluids such as saliva and is the primary cause of infectious mononucleosis (IM), but is also associated with other illnesses, such as certain types of lymphoma and nasopharyngeal carcinoma. EBV is one of the most prevalent human viruses, infecting around 95% of the population, and no particular therapy or vaccination is available [28]. Primary EBV infection in childhood is usually asymptomatic; nevertheless, when infection occurs later in life, the virus can cause IM in 35–50% of instances [29]. Human T-lymphotropic virus 1 (HTLV-1) is primarily transmitted through blood transfusions, sharing needles, and from mother to child during childbirth or breastfeeding, and chronic infection with HTLV-1 can led to a rare type of leukemia and a neurological condition called HAM/TSP [28].

Virus-mediated carcinogenesis involves a series of sequential steps that transform normal cells into cancer cells. These steps—initiation, promotion, and progression—have been extensively studied and documented [28,30]. During the initiation phase, a carcinogen interacts with the host DNA, setting the groundwork for subsequent cellular changes. Following initiation, the promotion stage ensues, characterized by the onset of cell proliferation. This phase can span from a few months to several years, depending on various factors. The final stage of virus-mediated carcinogenesis is tumor progression, which entails the spreading and development of the malignant tumor. Human oncoviruses employ direct and indirect mechanisms to transform cells, known as viral carcinogenesis [31]. In direct viral carcinogenesis, the virus incorporates genes that stimulate cellular growth and enhance resistance to apoptosis, thereby altering the DNA repair mechanism [26,32]. Consequently, tumor suppressors such as p53 and pRb are deactivated during viral oncogenesis until the DNA repair mechanism is restored. Failure to resume proper DNA repair mechanisms may trigger cell death [32]. It is important to note that viral infections can increase the susceptibility of our DNA to mutations. For instance, the viral antigen EBNA-1 derived from the Epstein–Barr virus (EBV) has been found to induce genomic instability by activating RAG1 and RAG2 [25,28]. EBV has been associated with various cancers, including Burkitt lymphoma (BL), Hodgkin disease, nasopharyngeal carcinoma (NPC), gastric cancer, T/NK lymphoma (nasal natural killer/T-cell lymphoma), as well as AIDS- or transplantation-associated lymphomas [33]. While conventional antiviral drugs like acyclovir and ganciclovir have demonstrated the ability to inhibit EBV lytic replication in laboratory settings, none have received FDA approval thus far [28,29] Furthermore, numerous antiviral treatments tested in clinical trials have proven ineffective [34]. As a result, there is a pressing need to develop innovative, effective, and safe antiviral medications targeting EBV and BL.

## 4. Treatment and Side Effects

### 4.1. HL

Treatment of HL generally involves a combination of chemotherapy and radiation therapy. Radiotherapy is typically used to destroy cancer cells that may remain after chemotherapy [35]. Involved-site radiation therapy (ISRT) targets only the affected lymph nodes or areas of the body rather than the entire lymphatic system [35]. This approach can reduce the risk of long-term side effects of radiotherapy. The side effects of radiotherapy for HL can include fatigue, nausea, and skin changes, as well as long-term effects such as an increased risk of developing other types of cancer [35,36].

In the treatment of HL, the standard therapeutic approach in the United States and many other countries is ABVD, which consists of a combination of doxorubicin, bleomycin, vinblastine, and dacarbazine [2]. Conversely, in Germany, the BEACOPP regimen has gained wide usage. BEACOPP incorporates bleomycin, etoposide, doxorubicin, cyclophosphamide, vincristine, procarbazine, and prednison [2]. Another treatment regimen known as Stanford V, which involves the administration of doxorubicin, vinblastine, mechlorethamine, vincristine, bleomycin, etoposide, and prednisone, is also utilized for HL [2]. Comparatively, these treatment regimens yield similar response rates. However, BEACOPP has demonstrated slightly superior survival rates when compared to ABVD and Stanford V. Nevertheless, this advantage comes at the cost of increased toxicity, including a heightened risk of developing secondary acute myeloid leukemia/myelodysplastic syndrome and, notably, sterility [2].

A review by Mondello, Musolino, Dogliotti, Bohn, Cavallo, Ferrero, Botto, Cerchione, Nappi, De Lorenzo, Martinelli, Wolf, Schmitt, Loseto, Cuzzocrea, Willenbacher, Mian and Straus [37] compared the efficacy and safety of ABVD and BEACOPPesc as first-line treatments for advanced-stage HL in a real-world setting. The retrospective analysis included 397 HL patients treated in seven European cancer centers from October 2009 to October 2018 [37]. The study found that BEACOPPesc achieved a higher rate of complete metabolic remission than ABVD, but also resulted in more frequent severe adverse events. Furthermore, the long-term outcome in terms of overall survival was similar between the two regimens. However, a trend towards superior progression-free survival in high-risk patients treated with BEACOPPesc was observed [37]. Although ABVD is an effective and less toxic therapeutic option for advanced-stage HL, BEACOPPesc offers better initial tumor control, but is associated with higher toxicity [37].

Because of the bulkiness of the illness or the persistence of positron emission tomography and computed tomography (PET/CT) positive scans following chemotherapy, systemic chemotherapy may be augmented with local radiation [2,38]. Bleomycin pulmonary toxicity is a problem, necessitating baseline pulmonary function tests and monitoring for the emergence of any symptoms along the route [2]. Doxorubicin, a widely used chemotherapy drug, has the potential for cardiac toxicity, which refers to heart damage or dysfunction [39]. The exact mechanism is not fully understood, but it involves oxidative stress and mitochondrial dysfunction [39]. The risk of cardiac toxicity with doxorubicin increases with higher cumulative doses and prolonged exposure [40]. Moreover, risk factors of doxorubicin cardiotoxicity include cumulative dose, age, pre-existing heart conditions, and concurrent cardiotoxic medications. Acute toxicity of chemotherapy can cause arrhythmias, while chronic toxicity can lead to heart failure or cardiomyopathy [40,41].

Immunotherapy, which involves using drugs that stimulate the immune system to attack cancer cells, has also been used for the treatment of HL. The most used immunotherapy drug for HL is brentuximab vedotin (Adcetris) [42]. It is an antibody–drug conjugate that combines an anti-CD30 monoclonal antibody with a chemotherapy agent [42]. CD30 is a protein expressed on the surface of HL cells, and brentuximab vedotin delivers the chemotherapy agent directly to these cells, leading to their destruction [42]. The other common type of immunotherapy for HL is checkpoint inhibitors, such as nivolumab (Opdivo) and pembrolizumab (Keytruda) [43]. These drugs block a protein called PD-1, which can help cancer cells evade detection by the immune system and prevent immune cells from attacking cancer cells [43]. While immunotherapy can effectively treat HL, it can also cause side effects such as fatigue, rash, and diarrhea. Additionally, some patients may experience more severe side effects, such as autoimmune disorders or inflammation of organs including the lungs or liver [36].

### 4.2. NHL

Treatment choice depends on several factors, including the stage and type of NHL, the overall health of the patient, and the potential risks and benefits of each therapy [44]. For many years, the standard chemotherapy treatment for patients with aggressive NHL has been cyclophosphamide, doxorubicin, vincristine (Oncovin), and prednisolone (CHOP) [45,46]. A comprehensive study performed 26 years ago revealed the spectrum of issues encountered by CHOP patients and the predicted occurrence and severity of side effects during treatment [46]. A 75-item self-report questionnaire was used to gather data at each treatment cycle, with the severity of each side effect assessed on a five-point scale in that study [46]. Nineteen subjects were given 99 cycles of CHOP and completed 74 questionnaires (75% response rate) and patients reported a total of 80 adverse events. The most prevalent issue was alopecia, with all patients suffering some hair loss by cycle 3. Fatigue was the second most prevalent adverse effect (incidence = 77%) followed by taste change (incidence = 74%). The early half of the therapy regimen was plagued by nausea and exhaustion. Moreover, patients rated post-chemotherapy nausea as the “most bothersome” issue, followed by weariness, taste changes, constipation, and difficulties sleeping [46]. These findings suggested that patients receiving CHOP had a wide range of issues, many of which warrant additional research.

Radiotherapy and immunotherapy are also utilized for NHL, in addition to chemotherapy. Both treatments have shown promising results in improving patient outcomes, but can also cause side effects such as fatigue, nausea, and immune-related toxicities [44]. Several types of radiotherapy and immunotherapy are currently used for NHL treatment, and the choice of therapy depends on the specific subtype and stage of NHL. Furthermore, the most common type of radiotherapy used for NHL is external beam radiation therapy, which delivers high-energy X-rays to the affected area, whereas total body irradiation delivers radiation to the entire body [44].

Multiple immunotherapy treatments are used for NHL, including monoclonal antibodies (mAbs), chimeric antigen receptor (CAR) T-cell therapy, and immune checkpoint inhibitors. For instance, the anti-CD20 mAb rituximab is commonly used in combination with chemotherapy for NHL and has been shown to improve patient outcomes. CAR T-cell therapy, which involves engineering a patient’s T cells to recognize and attack cancer cells, has also shown promising results in clinical trials for certain types of NHL [47]. Immune checkpoint inhibitors such as pembrolizumab and nivolumab are also being investigated for NHL treatment [48].

Collectively, the current literature demonstrates that most therapeutic options currently available for HL and NHL, although effective, have several side effects, which can even be life-altering in some cases. Therefore, more research is necessary to find effective and safer therapeutic alternatives for lymphoma patients.

## 5. Role of Gut Microbiota in Lymphoma

The microbiota encompasses diverse bacteria, fungi, eukaryotic viruses, archaea, and bacteriophages that coexist with the host, potentially offering mutual benefits [49]. Over the past two decades, extensive research has been conducted to unravel gut microbiota composition and therapeutic potential [10,50,51,52,53,54,55]. Furthermore, LPS, an endotoxin primarily present in the outer membrane of gram-negative bacteria such as *Escherichia coli* (*E. coli*), *Salmonella*, *Shigella*, and *Pseudomonas*, possesses the ability to trigger inflammation within the body. Imbalances or excessive proliferation of these bacteria within the gut microbiota can result in heightened LPS production, leading to inflammation and potentially contributing to carcinogenesis. Various factors, including dysbiosis, small intestinal bacterial overgrowth, leaky gut, and inflammatory bowel disease, can contribute to elevated levels of LPS in the gut. To mitigate LPS-related concerns, it is imperative to maintain a healthy gut microbiota by adopting a nutritious diet, regular exercise, and effective stress management techniques. These lifestyle measures are crucial for promoting optimal gut health and minimizing the potentially detrimental effects of elevated LPS levels.

A study by Mamgain et al. (2021) underlined the growing evidence suggesting that the microbiota plays a fundamental role in developing and progressing B and T-cell lymphomas [56]. Another study by Yuan et al. (2021) examined the gut microbiota of participants with untreated DLBCL and healthy volunteers. The analysis revealed that participants with untreated DLBCL had a distinct microbial composition compared to healthy individuals. Specific differences included a higher abundance of the *Escherichia-Shigella* genus and lower levels of certain metabolic pathways [57].

Ataxia telangiectasia (A-T) is a rare genetic disorder primarily affecting children, characterized by progressive dysfunction in multiple systems and associated with high lymphoid malignancies [58]. Neoplasia develops in about 30–40% of people with A-T during their lifetime, with NHL accounting for over 40% of these tumors, acute lymphocytic leukemias for around 20%, and HL for about 5% [59,60,61,62,63]. Cheema et al. (2016) investigated the impact of intestinal microbiota on various aspects of health, including nutrient metabolism, immune system modulation, obesity, and potential carcinogenesis, in two groups of mice: A-T mutated gene (Atm-deficient) and wild-type, which have different intestinal microbiota compositions due to their genetic differences [64]. The authors employed [64] a high-resolution mass spectrometry approach to analyze the metabolic profiles of urine and feces from both groups of mice. They discovered that the composition of the intestinal microbiota significantly influenced specific metabolic changes, potentially alleviating a glycolytic phenotype—a metabolic state associated with increased glucose metabolism. Moreover, they identified certain metabolites, such as 3-methyl butyrolactone, kynurenic acid, and 3-methyladenine, known for inhibiting cancer development, to be elevated in both Atm-deficient and wild-type mice with limited intestinal microbiota [64]. Similarly, another study was conducted on an A-T mouse model to investigate the relationship between intestinal microbiota and the development of B-cell lymphoma [65]. The researchers compared different isogenic mouse colonies with varying bacterial communities [65]. They found that the microbiota composition significantly influenced disease penetrance, latency, lifespan, molecular oxidative stress, and systemic leukocyte genotoxicity. The study employed high-throughput sequencing analysis to identify specific bacterial phylotypes associated with the mouse colonies [65]. One particular bacterium, *Lactobacillus johnsonii*, was deficient in the mouse colony that was more prone to cancer [65]. To test its impact, the authors conducted a short-term oral transfer of *L. johnsonii* and observed a reduction in genotoxicity [65]. The intervention led to a decrease in systemic genotoxicity, which was linked to reduced basal leukocyte levels and an inflammatory state mediated by cytokines. The findings suggested that restoring *L. johnsonii* or modifying the intestinal microbiota could be a potential translational intervention for individuals at risk of B-cell lymphoma or other diseases driven by genotoxicity or oxidative stress responses [65].

Another study by Yamamoto and Schiestl (2014) discussed the association between certain bacteria and the development of mucosal-associated lymphoid tissue (MALT) lymphoma, which originates in the marginal zone [66]. Approximately 90% of MALT lymphomas are associated with *Helicobacter* infection, elimination of which has been shown to achieve complete remission in around 80% of cases [66]. The causative effect of *Helicobacter* in MALT lymphoma development has been demonstrated in animal models [66]. Mice infected with *Helicobacter felis*, a close relative to *H. pylori*, developed lymphoepithelial lesions associated with MALT lymphoma. Similarly, gerbils infected with *H. pylori* showed increased gastritis and intestinal metaplasia [66]. These animal models have been used to study the mechanisms, disease progression, and regression of *H. pylori* infections. Likewise, *Helicobacter helmanii*, found in humans and mice, has been also shown to contribute to the development of MALT lymphoma [66]. In animal models, *H. helmanii*-induced lymphoma is preceded by inflammation and high endothelial venule-like vesicles, which are associated with lymphocyte recruitment and found in other chronic inflammatory conditions [66]. Although associations between other bacteria, such as *Campylobacter jejuni*, *Borrelia burgdorferi*, *Chlamydia psittaci*, and *Streptococcus bovis,* and lymphoma have been observed in humans, the role of these bacteria in lymphoma development is not yet fully understood [66]. Animal models could provide valuable insights into the etiology, progression, and treatment of microbe-associated lymphomas [66].

Emerging evidence has elucidated the role of gut microbiota in protecting from pathogens; maintaining metabolic, endocrine, and immune functions; and modifying drug action and metabolism [10]. Gut microbial metabolites, including SCFAs, inosine, urolithin A, urolithin B, and bacteriocins, have shown a broad spectrum of biological activities in previous studies, including anticancer and immunomodulatory functions [67,68,69]. Dysbiosis in the gut microbiota is believed to lead to chronic inflammation and immune dysregulation, both known risk factors for lymphoma [70]. Alterations in the composition and diversity of the gut microbiota have been associated with an increased risk of cancer [71]. For instance, a study found that participants with diffuse large B-cell lymphoma had a lower diversity of gut bacteria than healthy controls. Similarly, another study found that gut microbiota dysbiosis was linked to an increased risk of NHL [72]. Individuals with NHL had less diverse gut microbiota than healthy individuals, with a decrease in the abundance of certain bacterial species associated with anti-inflammatory properties, such as *Faecalbacterium* and *Bifidobacterium* [73]. Another study found that a higher diversity of gut bacteria was associated with a lower risk of lymphoma, while antibiotic use correlated with an increased risk [74].

In addition to the gut microbiota, the oral microbiota has also been implicated in lymphoma risk. For instance, individuals with oral infections, such as periodontitis, were found to have a higher risk of developing lymphoma [75]. Although the mechanisms linking microbial communities to lymphoma risk are not fully understood, these findings suggested that alterations in certain microbial communities may contribute to the development of lymphoma. It is essential to note that the relationship between gut microbiota and lymphoma is an area of active research, and more studies are needed to fully comprehend its clinical implications.

### 5.1. Prebiotics

Prebiotics are non-digestible food fibers that selectively stimulate the growth and activity of beneficial bacteria in the gut. Prebiotics have been studied for their potential impact on cancer and immune function. Although direct research on the role of prebiotics in lymphoma is limited, evidence suggests that prebiotics may indirectly influence lymphoma development and progression by modulating the gut microbiota and immune responses. Several studies have explored the interaction between prebiotics, gut microbiota, and cancer, highlighting their potential implications for lymphoma [76]. Delzenne and Cani (2011) discussed the interplay between obesity, gut microbiota, and metabolic disorders, emphasizing the potential of prebiotics in modulating gut microbiota composition and metabolic health [77]. This modulation may indirectly impact lymphoma development [77]. Uccello et al. (2012) investigated the interaction between gut microbiota and colorectal cancer, emphasizing the potential of prebiotics in promoting the growth of beneficial bacteria and modulating the gut environment [78]. Gentile and Weir (2018) discussed how prebiotics can shape gut microbiota composition and function, influencing systemic inflammation, immune responses, and potentially cancer development, including lymphoma [79]. Tuohy et al. (2012) highlighted how prebiotics can promote the growth of beneficial bacteria, enhance the production of SCFAs, and improve gut barrier function, all of which may have implications for lymphoma and immune health [80]. Furthermore, Rattanathammethee et al. (2020) studied the impact of chemotherapy on gut microbiota dysbiosis in patients with acute myeloid leukemia and febrile neutropenia [81]. Prebiotics have been shown to be beneficial in mitigating dysbiosis and maintaining a healthy gut environment, which could be relevant to people with lymphoma undergoing chemotherapy. In summary, while there is no direct research on the role of prebiotics in lymphoma, further investigations are needed to fully understand the potential benefits of prebiotics in lymphoma treatment and prognosis. Understanding the interplay between prebiotics, gut microbiota, and lymphoma may provide new insights and therapeutic opportunities for this complex disease.

### 5.2. Probiotics

Probiotics are live microorganisms that confer health benefits when consumed in adequate amounts and play a fundamental role in maintaining the balance and diversity of the gut microbiota as well as its metabolites. Studies have demonstrated that specific strains of probiotics can modulate the composition of gut microbiota by increasing the levels of beneficial bacteria, such as *Bifidobacterium* and *Lactobacillus*, while reducing the population of potentially harmful bacteria (Sanders et al. [82]). Probiotics also enhance gut barrier function by promoting the production of tight junction proteins and mucus, strengthening the intestinal barrier (Resta-Lenert and Barrett [83]). Furthermore, they have immunomodulatory effects, stimulating the production of anti-inflammatory compounds and regulating immune cell activity (Round and Mazmanian [84]). Probiotics can also influence host metabolism by producing metabolites such as SCFAs, which benefit gut health (Ríos-Covián et al. [85]). They can reduce the growth and activity of pathogenic microorganisms in the gut through antimicrobial substances and competition for nutrients (Hill et al. [86]). While a growing body of research explores the interrelationship between gut microbiota and cancer, these studies primarily focus on colorectal cancer and other solid tumors [87]. The role of probiotics in lymphoma specifically has yet to be extensively investigated. However, it is worth noting that individuals undergoing cancer treatments, such as chemotherapy and radiation therapy, often experience gastrointestinal side effects, including diarrhea and alterations in gut microbiota [88,89]. In these cases, probiotics have been studied as a supportive measure to manage the treatment-related side effects [87]. Some studies have suggested that certain strains of probiotics may help to reduce the severity and duration of diarrhea in people with cancer [89,90].

### 5.3. Postbiotics

Postbiotics (also known as gut microbial metabolites) are a term used to describe the by-products or metabolic compounds produced by gut bacteria during their growth and fermentation process. These by-products include various substances such as SCFAs, enzymes, organic acids, peptides, polysaccharides, vitamins, and other metabolites. Postbiotics have gained increasing attention recently due to their potential health benefits and role in modulating gut microbiota.

Postbiotics derived from probiotic bacteria have been found to exert several beneficial effects on the gut microbiota and overall health [91]. They can influence the gut microbiota in several ways, including the modulation of microbial composition [91] and enhancement of gut barrier integrity. Strengthening the intestinal barrier function is critical in preventing the passage of harmful substances from the gut into the bloodstream. This helps maintain gut integrity and reduces the risk of inflammation and immune activation [92]. Postbiotics can help promote the growth of beneficial bacteria and inhibit the growth of harmful or pathogenic bacteria, thereby improving the overall microbial composition [92]. They can promote the production of anti-inflammatory cytokines and enhance the activity of immune cells, helping to regulate immune function and reduce inflammation [93]. Postbiotics including SCFAs, are produced through the fermentation of dietary fiber by the probiotic gut bacteria [94]. SCFAs provide an energy source for the colonocytes (cells lining the colon) and have anti-inflammatory properties [93]. They also influence gene expression, metabolism, and satiety signaling [93]. Furthermore, some postbiotics exhibit direct antimicrobial activity against pathogenic bacteria, helping to control their growth and reducing the risk of infections [95].

K. Inamura (2021) examined the potential of postbiotics against lymphoma cells. The researcher used heat-killed *Lactobacillus casei*, a type of probiotic bacteria, and its postbiotic metabolite, lactate, in a mouse lymphoma model [96]. The results suggested that heat-killed *L. casei* and lactate could enhance the immune response against lymphoma cells, reducing tumor growth [96]. Moreover, the authors investigated various molecular pathways involved in the interaction between the gut microbiota and the immune response against cancer. The molecular pathways related to pattern recognition receptors (PRRs), toll-like receptors (TLRs), SCFAs, immune checkpoint pathways, and cytokines and chemokines were investigated [96]. Another study by Gill, PA et al., 2018 investigated the effects of SCFAs on the immune response to melanoma in a mouse model. The authors found that SCFAs, particularly butyrate and propionate, enhanced the anticancer immune response by promoting the activation and function of immune cells [97]. This suggested that SCFAs may have potential immunomodulatory effects in cancer. While these studies provide some preliminary evidence of the potential immunomodulatory effects of postbiotics in cancer, including some preliminary observations against lymphoma, further research is needed to establish their efficacy, safety, and molecular mechanisms of action. Table 1 shows the studies currently available in the literature on the role of postbiotics against lymphoma.

#### 5.3.1. SCFAs

SCFA production occurs primarily in the large intestine through the fermentation of undigested starch and non-starch polysaccharides [106]. These SCFAs, namely, acetic (acetate), propionic (propionate), and butyric (butyrate) acids, are short monocarboxylic acids [106,107]. While acetate is minimally oxidized in the liver [108], colonocytes metabolize SCFAs, and the remaining unmetabolized portions enter the portal circulation to act as an energy source for hepatocytes [108]. Given that only a small fraction of SCFAs from the colon enter the systemic circulation, fecal concentration has been employed to indicate SCFA production in the colon [10]. Numerous studies have revealed the beneficial effects of SCFAs in various conditions, including diabetes, cancer, and hypertension [109,110,111].

Butyric acid and other SCFAs have been found to change gene expression in human cancer epithelial and lymphoid cells, including Raji and Rael cell lines, which are derived from Burkitt’s lymphoma and carry EBV genomes in latency type III and I, respectively [98]. Moreover, propionate was found to inhibit the growth of T-lymphoma cells in vitro and in vivo in mice, indicating its potential implementation in lymphoma therapy [99]. Similarly, another study revealed that butyrate enhanced the anti-tumor effects of 5-fluorouracil (5-FU) in mice with colorectal cancer by increasing tumor cell death and reducing tumor growth [11]. Moreover, 5-FU may be used in combination with other chemotherapy drugs as part of a treatment regimen [112,113] although it is not considered a first-line therapy for lymphoma. Wanget al. [11] also revealed that butyrate modulated the activity of the transforming growth factor-beta 1 TGF-β1/Smad3 signaling pathway, which is a cytokine that plays a crucial role in regulating cell growth, differentiation, apoptosis, and immune response. It exerts its effects by binding to cell surface receptors, activating downstream signaling pathways, including the Smad proteins [114]. The TGF-β1/Smad3 signaling pathway is indeed relevant to lymphoma, and its dysregulation can contribute to tumor development and progression [114]. However, further research is needed to confirm these findings and to determine the interactions of butyrate with standard chemotherapy [11], as well as its impact on lymphoma. Contradictory findings regarding the effects of SCFAs on lymphoma have also been reported.

The antiproliferative action of the SCFAs butyrate, propionate, isobutyric acid, and acetic acid has also been reported against human gastric (Kato III) and colon cancer (Caco-2, DLD-1, and WiDr) cells, with butyrate being more active than its counterparts [115,116,117,118,119]. These investigations further revealed that the antiproliferative effect of SCFAs was mediated via modification of the cell cycle, DNA replication, recombination, and repair, as well as apoptosis. Sodium butyrate (the sodium salt of butyric acid) was also found to promote DAPK expression, which resulted in apoptosis via lowering FAK protein levels in AGS and MKN45 human gastric cancer cells [120]. Another study by Kobayashi, Mikami, Uwada, Yazawa, Kamiyama, Kimura, Taniguchi and Iwano [121] demonstrated that the SCFA propionate enhanced the cytotoxic effect of cisplatin by regulating GPR41 signaling pathways in the HepG2 liver cancer cells [121]. Overall, SCFAs can regulate several molecular pathways in cancer and normal cells, and further research is required in order to fully comprehend the direct and indirect effects of SCFAs on lymphoma.

**Figure 2:**
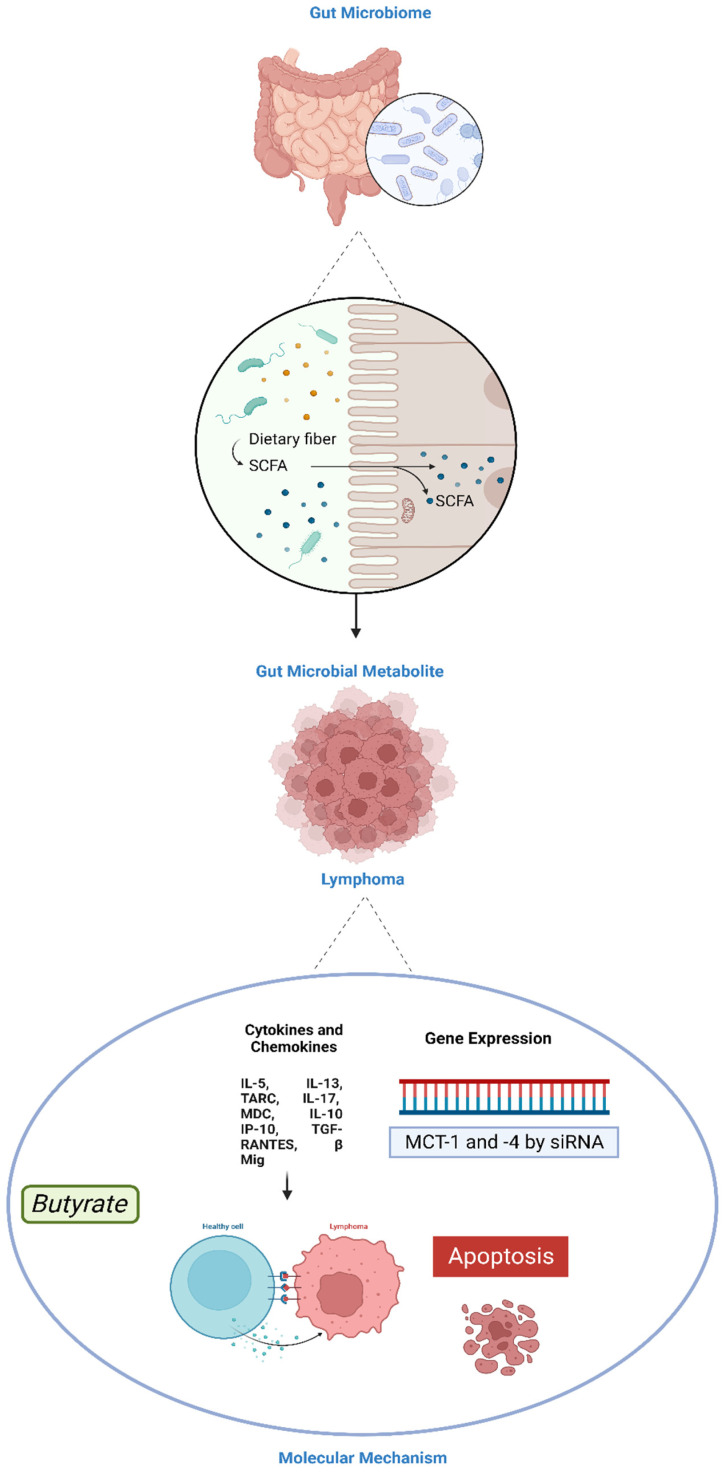
The interaction between short-chain fatty acids (SCFA) and lymphoma.

Among the three major SCFAs, several reports have explored butyrate in relation to lymphoma. For example, a study by Perrine et al. (2007) investigated the effects of butyrate on the growth and differentiation of Burkitt’s lymphoma cells [122]. The authors found that butyrate treatment inhibited cell proliferation and induced cell differentiation in the Akata lymphoma cell line [122] (Figure 2).

#### 5.3.2. Bacteriocins

Bacteriocins, which are small amphiphilic peptides produced by archaea and bacteria, have garnered significant research attention [28,69]. Among the bacteriocins synthesized by lactic acid bacteria, several exhibit bactericidal or bacteriostatic effects on bacterial strains that are similar or closely related [123]. These bacteriocins can be classified into two classes: Class I, known as lantibiotics, and Class II, referred to as non-lantibiotics [69]. Their mode of action involves creating pores in the cell membrane, leading to a decrease in intracellular pH and the efflux of small metabolites [69]. Class I bacteriocins, or lantibiotics, encompass single peptides such as nisin, mersacidin, and lacticin. Class II bacteriocins, or non-lantibiotics, include pediocin, lactacin, and lactococcin. Although the precise mechanisms of action for each bacteriocin remain elusive, their ability to bind to lipid II, the primary transporter of peptidoglycans from the cytoplasm to the cell wall, has been identified as an effective mechanism in various studies [69]. Evidence suggests that bacteriocins play a crucial role in the probiotic efficacy of the gut microbiota [28,124,125,126]. While the antimicrobial properties of bacteriocins produced by probiotic lactic acid bacteria are well-established, their preventive and therapeutic roles in cancer are not yet fully elucidated. Further research is needed to comprehensively understand the potential impact of bacteriocins in the context of cancer prevention and treatment.

Nisin, an extensively studied bacteriocin, has demonstrated potential as an anticancer agent, supported by several investigations conducted on cancer cells. Nisin is produced through bacterial fermentation and is a polycyclic peptide renowned for its antibacterial properties, particularly against a broad spectrum of Gram-positive bacteria such as *Staphylococcus aureus* and *Listeria monocytogenes* [127]. In recent years, research efforts have also focused on exploring nisin’s potential as a peptide with anticancer properties, particularly in colorectal cancer cells [128,129,130].

Nisin has been licensed as a food preservative in over 50 countries and is generally recognized as safe for humans by the World Health Organization [101]. Recent research has reported that nisin induced apoptosis and inhibited the growth of Jurkat lymphoma cells [101]. Moreover, the IC_50_ value of nisin against the Jurkat cell line was found to be 225 mM; however, the same concentration was also found to be toxic to human lymphocytes in that study. The authors suggested that nisin may have potential as an adjuvant therapy for lymphoma [101]. It has also demonstrated its ability to impede tumorigenesis in head and neck squamous carcinoma cells both in vitro and in vivo [129]. In these studies, the concentration of a particular nisin variant (nisin ZP and nisin AP) exhibited a direct relationship with the induction of apoptotic cancer cell death and a reduction in cell proliferation among head and neck cancer cells [129]. Furthermore, nisin has been observed to enhance the apoptotic index in various cancer cell lines by activating the intrinsic apoptotic pathway [128]. The anticancer activity of a bacteriocin called enterocin CRL35 was also evaluated against human lymphoma cells, and it was found to induce apoptosis in a Dalton’s-lymphoma-bearing ascites mice model and significantly inhibit their growth [102,131]. The authors concluded that enterocin CRL35 can be a potential therapeutic agent against lymphoma [102]. Furthermore, it was previously claimed that nisin negatively affected cancer cells via CHAC1 (ChaC Glutathione Specific Gamma-Glutamylcyclotransferase 1), a pro-apoptotic cation transport regulator and apoptotic mediator in carcinogenesis [132]. Nisin-induced CHAC1 expression, in turn, increased calcium influxes and caused G_2_ cell cycle arrest, which resulted in apoptosis and decreased tumor cell proliferation [132]. Nevertheless, the authors stated that the ideal therapeutic dosage for the possible use of nisin in cancer therapy must be found [132].

Earlier research also explored the antiviral activities of bacteriocins against several viruses, including the oncogenic viruses- Herpes simplex virus (HSV)-1 and HSV-2 [133,134,135,136]. However, the activity of bacteriocins against viruses that cause lymphoma, including EBV, remains to be investigated and understood.

#### 5.3.3. Inosine

Inosine (a nucleic acid base), a crucial component in purine metabolism, is generated by particular RNA deaminases deaminating adenosine [137]. Studies have explored its potential benefits for enhancing athletic performance as a dietary supplement and its possible medical applications for treating certain conditions [9]. Currently, limited research is available on the effects of inosine on lymphoma. Some studies have suggested that it may impact the immune system [9], which could make it a beneficial adjunct therapy for treating certain types of cancer, including lymphoma (Table 1).

In a recent in vivo study, the intestinal probiotic bacterium *Bifidobacterium pseudolongum* enhanced the immunotherapy response against four mouse models of cancer (colorectal cancer, intestinal cancer, bladder cancer, and melanoma) through the production of inosine, which was dependent on the T-cell expression of the adenosine A2A receptor [111].

Isoprinosine (IP), also known as inosine pranobex or methisoprinol, is a compound composed of inosine and pranobex, which is a combination of dimethyl amino isopropanol (dimepranol) and p-acetamidobenzoate (acedoben) [28,68]. Since 1971, IP has been extensively utilized in treating various viral diseases, including HSV, Human papillomavirus (HPV), HIV, cytomegalovirus, influenza, acute respiratory infections, and EBV infections, owing to its immunomodulatory properties and favorable safety profile. IP has shown efficacy in enhancing the normal immune response of lymphocytes when administered after the onset of viral infections [28,68]. It is believed that IP acts as an immunomodulator in viral infections by increasing the levels of pro-inflammatory cytokines such as IL-2 and INF-c in mitogen- or antigen-activated cells, thereby promoting T-lymphocyte differentiation and inducing lymphoproliferative activity [28,138,139]. Additionally, through its direct action and in conjunction with INF-c, IP has been shown to decrease the production of anti-inflammatory cytokines like IL-10, suggesting its immunomodulatory effects on innate and adaptive immunity. IP has also been reported to enhance the population and activity of natural killer cells, as well as potentiate phagocytosis and macrophage chemotaxis [28,140,141,142].

The in vivo antiviral activity of IP was evaluated against murine gamma herpesvirus 68 (MHV68), a natural pathogen of mice commonly used as a model for EBV infection. Following a two-week treatment period, IP administration increased virus-neutralizing antibodies, leukocytes, and neutrophils [100]. However, the antiviral effect was transient and dissipated within 120–150 days. Tumor formation in the MHV-infected group was 7.5% after IP treatment, compared to 17.5% in the untreated group. The authors suggested that repeated IP injections may be necessary for persistent EBV infections [100]. Further investigations are required to understand the potential benefits and jeopardies of inosine in lymphoma treatment [9].

#### 5.3.4. Urolithins

Urolithins, which are derivatives of dibenzo[b,d]pyran-6-one, are produced by the human gut microbiota through the metabolism of ellagitannins and ellagic acid (EA) [143]. Natural sources contain various forms of urolithin, including urolithin A (UA), urolithin B (UB), urolithin C (UC), and urolithin D (UD). Among these, UA has been extensively studied and is commonly detected in human feces and urine [143]. EA, a secondary metabolite present in diverse foods such as seeds (walnuts, almonds), fruits (persimmons, berries, peaches, palms, and pomegranate), and vegetables (punicalagin, corilagin, and vescalagin), possesses antioxidant, anti-inflammatory, neuroprotective, and anticancer properties [103]. These anticancer effects of EA are attributed to its ability to influence apoptosis, cell proliferation, and cell cycle regulation. Furthermore, EA metabolites, including urolithins, have demonstrated potential anticancer properties [103].

**Figure 3:**
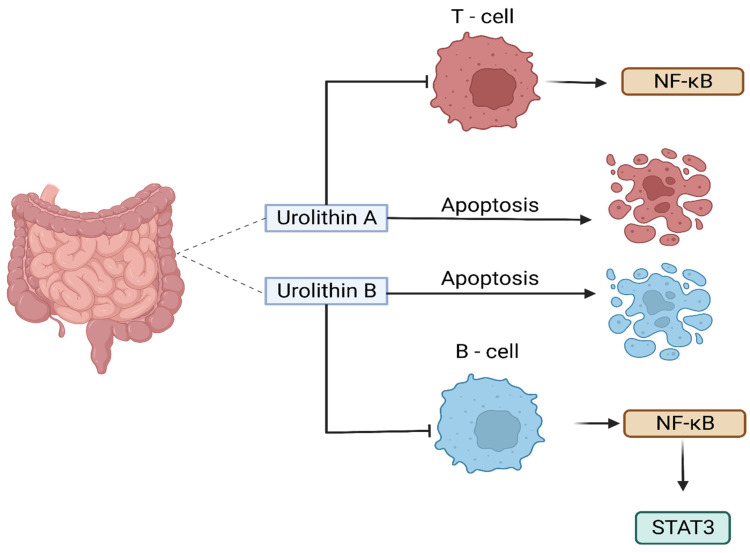
The key effect of urolithin A and urolithin B against T-cell lymphoma and B-cell lymphoma is through the NF-κB signaling pathway.

UA, in particular, has been investigated for its role in modulating carcinogenesis, apoptosis, DNA damage caused by oxidative stress, and angiogenesis in various malignancies [144]. A recent study examined the effects of UA on human anaplastic large lymphoma cell lines (KARPAS-299 and MAC-2A), as well as human leukemia cell lines (MOLT-4 and HL-60), and found that UA inhibited the growth of lymphoma cells and induced apoptosis (Figure 3) [103]. The authors also found that UA inhibited the activation of the NF-κB signaling pathway, which is involved in the survival of lymphoma cells [103]. The inhibition of the NF-κB signaling pathway and the activity of an enzyme called STAT3 by urolithin B (UB) in B-cell lymphoma was also reported by Lv et al. [104], which led to the growth inhibition and induction of apoptosis in lymphoma cells (Figure 3). Similarly, another study reported that UA and its derivatives inhibited the growth of T-cell lymphoma and induced apoptosis through the inhibition of the Akt enzyme activity, which is involved in cell survival and proliferation [105].

**Figure 4:**
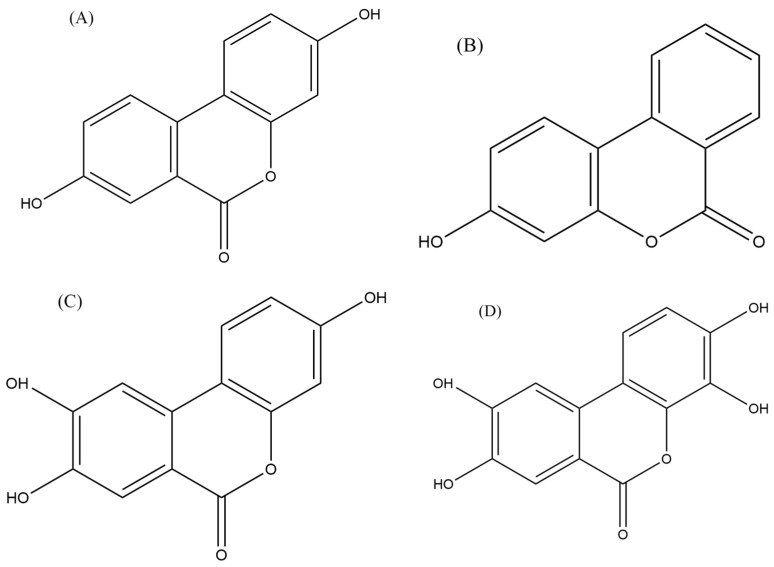
Chemical structure of urolithins (**A**–**D**).

Collectively, these studies suggest that urolithins, particularly UA and UB (Figure 4), may have anti-lymphoma properties. However, further studies are needed to confirm these findings in a wide range of lymphoma cell lines as well as animal models to determine the optimal dose and in-depth mechanisms of action against lymphoma.

## 6. Conclusions and Future Directions

The role of the gut microbiota and gut microbial metabolites in lymphoma is an emerging field of research with significant implications for our understanding of the disease. Dysbiosis and alterations in the production of gut microbial metabolites have been found to be associated with lymphoma development. SCFAs derived from dietary fiber fermentation have shown potential in promoting regulatory T-cell function, suppressing inflammation, and preventing lymphoma. Similarly, EA-derived UA and UB have demonstrated the ability, albeit limited, to inhibit the growth of cancer cells and induce apoptosis in various cancer types, including lymphoma and leukemia. Conversely, LPS released due to dysbiosis can increase lymphoma risk through chronic inflammation.

Overall, studies on the clinical implication of gut microbiota and its metabolites in lymphoma are limited in the current literature. This article emphasizes the need for further investigation into the molecular mechanisms of gut microbial metabolites against lymphoma. Understanding how specific gut microbial metabolites modulate immune responses and influence lymphoma development will provide valuable insights into potential therapeutic strategies. Additionally, exploring the interactions between gut microbial metabolites and standard chemotherapeutic drugs could reveal synergistic effects, leading to more effective treatment options. Future directions for this research should involve comprehensive studies to evaluate and understand the mechanistic role of key gut microbial metabolites against lymphoma using various in vitro and in vivo models. In addition to therapeutic applications, preventive strategies against lymphoma targeting the gut microbiota and gut microbial metabolites should be explored. Investigating dietary interventions, such as prebiotics and probiotics supplementation to modulate the gut microbiota and promote the production of beneficial gut microbial metabolites, could offer potential preventive approaches for lymphoma in the future.

## Figures and Tables

**Table 1:** The mechanisms of the gut microbiome metabolites and/or chemotherapy against cancer/lymphoma.

Cell Type/Cancer	Gut Microbial Metabolites	Study Type	Mechanisms	Reference
Epithelial and lymphoid cells (Raji and Rael)	Butyric acid—SCFA	In vitro	The administration of nisin resulted in the stimulation of inflammatory and apoptotic reactions within tumor cells. The activation of the n-butyric gene was observed to decline when the cell membrane transporters MCT-1 and MCT-4 were downregulated through siRNA.	Astakhova et al. [98]
T-lymphoma cells	Propionate—SCFA	In vitro and in vivo	Inhibited the growth of T-lymphoma cells.	Mukovozov et al. [99]
Epstein–Barr virus (EBV)—lymphoma	Isoprinosine (IP)—Inosine complex	In vivo	After two weeks of treatment, IP resulted in elevated levels of virus-neutralizing antibodies, leukocytes, and neutrophils	Janíčková et al. [100]
Jurkat lymphoma cells	Nisin—Bacteriocins	In vitro	Induced apoptosis and inhibited their growth.	Kaur and Kaur [101]
Lymphoma cells	Enterocin CRL35—Bacteriocins	In vivo	Induced apoptosis in Dalton’s lymphoma-bearing cells and significantly inhibited their growth.	Baindara et al. [102]
Human anaplastic large lymphoma cell lines (KARPAS-299 and MAC-2A) and human leukemia cell lines (MOLT-4 and HL-60)	Urolithin A (UA)	In vitro	Inhibited the growth of lymphoma cells and induced apoptosis. Inhibited the activation of the NF-κB signaling pathway, which is involved in the survival of lymphoma cells.	Okumura et al. [103]
B-cell lymphoma	Urolithin B (UB)	In vitro	Inhibited the NF-κB signaling pathway and the activity of an enzyme STAT3, which lead to the growth inhibition and induction of apoptosis in lymphoma cells.	Lv et al. [104]
Human T-cells lymphoma	UA	In vitro	Inhibited the growth of lymphoma cells and induced apoptosis through the inhibition of the Akt enzyme activity, which is involved in cell survival and proliferation.	Lu et al. [105]

## Data Availability

No new data was created or analyzed in this study. Data sharing is not applicable to this article.
